# Transplantation of autologous cultivated oral mucosal epithelial sheets for limbal stem cell deficiency at Siriraj Hospital: a case series

**DOI:** 10.1186/s13256-022-03502-8

**Published:** 2022-08-04

**Authors:** Wipawee Booranapong, Panida Kosrirukvongs, Sunisa Duangsa-ard, Kanda Kasetsinsombat, Khanit Sa-ngiamsuntorn, Adisak Wongkajornsilp

**Affiliations:** 1 Department of Ophthalmology, Faculty of Medicine Siriraj Hospital, Mahidol University, 2 Wanglang Rd., Bangkoknoi, Bangkok, 10700 Thailand; 2 Department of Pharmacology, Faculty of Medicine Siriraj Hospital, Mahidol University, 2 Wanglang Rd., Bangkoknoi, Bangkok, 10700 Thailand; 3 Department of Biochemistry, Faculty of Pharmacy, Mahidol University, 447 Sri-Ayuthaya Rd., Rajathevi, Bangkok, 10400 Thailand

**Keywords:** Oral mucosal epithelial sheet, Limbal deficiency, Corneal replacement

## Abstract

**Background:**

The loss of limbal stem cells owing to either corneal burn or inflammation leads to the repopulation of opaque skin over the raw surface of the cornea. It has been proposed that reconstitution of oral mucosal stem cells over this raw surface will mimic the limbal stem cells and restore vision. The efficacy and safety of applying a sheet of cultivated oral mucosal cells as an autologous graft for corneal replacement were evaluated.

**Case presentation:**

The study was conducted during 2014–2015 and involved a total of six patients, of whom three had suffered a chemical burn and three had Stevens-Johnson Syndrome (SJS). Oral mucosal tissue was dissected from each patient, seeded onto irradiated J2 fibroblast feeder cells for 14 days, and analyzed for quality and safety 1 day before being transplanted onto the cornea of the affected eyes. After transplantation, topical antibiotic and anti-inflammatory eye drops were instilled four times daily, and the patients wore contact lenses. Subjects were clinically followed for visual acuities and adverse effects at 2, 4, and 6 weeks, 3 and 6 months, and 1 year post-transplantation. Data were presented descriptively. Visual acuities in patients improved at 2 weeks post-surgery. However, two patients with SJS had corneal ulcer at 2 weeks postoperatively. At the 1-year postoperative examination, the eyes of two patients were in good condition with decreased vascularization and epithelial defect.

**Conclusions:**

Cultivated oral mucosal epithelial sheet transplantation in limbal stem cell deficiency had a favorable efficacy. In this study, patients with chemical burn had more clinical benefit than those with SJS.

*Trial registration *ClinicalTrials.gov: NCT02415218. Registered retrospectively 4 Apr 2015 (https://clinicaltrials.gov/ct2/show/NCT02415218).

## Background

Management of limbal stem cell deficiency (LSCD) due to Stevens Johnson–Syndrome (SJS) or chemical burn to preserve vision is challenging. In Thailand, the treatment of penetrating keratoplasty is particularly cumbersome and difficult, with few eye donations. Patients who may receive allogeneic transplants often suffer eventual conjunctivalization, graft failure, and blindness. Success rates of 20% at 16 months and 27.3% at 36 months have been reported in the few studies performed [1, 2]. Complications, such as infection and liver and kidney injury, have been reported due to the use of long-term immunosuppressive medications [3–7]. Cultivated autologous oral mucosal epithelial sheet is a cell sheet that can be grafted onto the corneal stroma, replacing the corneal epithelium [8–13]. The presence of transparent mucosal stem cell in the mucosal sheet may restore the corneal surface with intact visual functions and circumvent allogeneic complications [14–16]. The purpose of the present study was to evaluate the efficacy and safety of cultivated oral mucosal epithelial sheet transplantation in patients with total LSCD.

## Case presentation

### Subjects

Six patients are reported here, of whom three suffered chemical burn and three had SJS. Of these six patients, three were men, with four right eyes affected. The mean age was 46.2 (range 34–66) years. All patients were prospectively enrolled as a single group and gave informed consent to the investigators at the Faculty of Medicine Siriraj Hospital (Institute Review Board [IRB] Ethical Approval No. SI 227/2013). The protocol could be terminated at any time due to loss to follow-up, inability to obtain mucosal tissue, or patient’s request. The inclusion criteria included patients aged > 20 years with total LSCD or total conjunctivalization from any cause. The exclusion criteria included pregnancy, severe infection, keratitis, and eye or buccal mucosal complications. All standard preparations, including surgery, work flow, laboratory tests, environmental controls, materials, chemical materials, records, pharmacological storage, quality/safety checks, and transportation, were managaged by Siriraj Hospital and complied with GMP guidelines for medicinal products. All procedures strictly followed the regulations in the U.S. Food and Drug Administration Code of Federal Regulations (CFR) Title 21 Part 1271 (Human cells, tissue and cellular and tissue-based products; https://www.accessdata.fda.gov/scripts/cdrh/cfdocs/cfcfr/CFRSearch.cfm?CFRPart=1271) and Title 21 CFR Part 600 (Biological product, current good tissue practice [CGTP]; https://www.accessdata.fda.gov/scripts/cdrh/cfdocs/cfcfr/CFRSearch.cfm?CFRPart=600), as well as with additional requirements for manufacturers of human cells, tissues and cellular and tissue-based products. Any adverse event, deviation, or intervention would be spontaneously reported to the IRB for review and further actions.

### Feeder cells

3T3-J2 fibroblasts were used as feeder cells. They were seeded to tissue culture flasks and maintained in Dulbecco's Modified Eagle Medium and 10% fetal bovine serum for 7–14 days to reach confluence, following which the 3T3-J2 cells were irradiated twice with 34 cGy X-ray each time to inhibit cell division. The irradiated cells (11.9 × 10^4^ cells/mL) were plated in 2-mL aliquots onto Nunc UpCell 3.5-cm dishes (Thermo Fisher Scientific, Waltham, MA, USA) or as 4.7-mL aliquots onto 60-mm tissue culture dishes and kept overnight prior to the seeding of mucosal epithelial cells.

### Oral mucosal sample collection

About 12–14 days before the planned transplant, an oral mucosal tissue sample (0.5 × 1.5 × 0.3 cm; width × length × depth) was excised under local anesthesia using disposable blade. The wound was sutured with Dacron 5-0 fiber to stop bleeding. Antibiotic and pain-relief medication were provided for 3 days. The mucosal tissue was soaked in 10% betadine in distilled water (1:8 dilution) for 10 min, then rinsed with 0.5% levofloxacin before being transported in 30 mL FDM (DMEM, 10% FBS, 0.125 μg/mL amphotericin B, 100 IU/mL penicillin G, 100 μg/mL kanamycin) in a sterilized box to the tissue culture facility. The dissected tissue was exposed to 1000 PU/mL dispase to separate the mucosal epithelial layer from the subcutaneous layer. The epithelial layer was digested with 0.25% trypsin EDTA to segregate epithelial cells. The isolated epithelial cells were seeded onto the irradiated feeder cells at a density of 2–3 × 10^5^ cells/35-mm dish and maintained in 2 mL KCM medium with epidermal growth factor at 37 °C, 5% CO_2_ for 12–14 days before the transplant. For the colony-forming assay (CFA), the epithelial cells were seeded at 3000–5000 cells/60-mm dish; the mucosal epithelial sheets were then examined for quality and safety (Table 1), including by immunohistochemistry, flow cytometry, and real-time PCR. Samples were submitted for sterility tests including bacterial culture, fungal culture and endotoxin assay at the Department of Microbiology, Siriraj Hospital.

**Table 1:** The criteria for the safety test of the epithelial sheet

Test	Method	Regulation
Viability	Trypan blue assay	> 80%
Endotoxin	LAL assay	< 0.5 EU/mL
Sterility test	Bacterial and fungal culture	No growth
Phenotype	Flow cytometry	–

*LAL* Limulus amebocyte lysate

### Transplantation

The mucosal sheet was transferred in a close-system box kept at 20 °C to the operating room. The cell sheet was harvested on a ring of PVDF membrane (outer diameter: 25 mm; inner diameter: 15 mm). The transplantation of the cultivated oral mucosal epithelial sheet was performed at 20°C with the patient under general anesthesia. The first step was the excision of conjunctiva and fibrous tissue on the cornea, namely symblepharon lysis, prior to the actual transplant. Before and after surgery, the subjects received topical antibiotic every 2 hours, and topical corticosteroid 4 times daily. Intravenous ceftriaxone (1 g) was given every 12 hours for 3 days, then oral antibiotic for the next 5 days. Intravenous solumedrol (125 mg) was given every 12 hours for the first day and once daily for 2 days, and oral prednisolone (25 mg) was given twice daily for 2–4 weeks. The subjects wore protective contact lenses for 1 year following surgery. The symptoms, clinical findings of inflammation, and the accompanying images were recorded in a dedicated hard disk and graded for severity at the follow-up periods: 2 and 4 weeks and 3, 6, and 12 months. The main outcome included corneal epithelial defect (0–100%) and conjunctivalization on the cornea (25–100%) within 1 year (graded from 0–5, with 0 = bad and 5 = excellent). The secondary outcome included visual acuity, corneal opacity, corneal vascularization, and complications. Only the investigators could access the collected data.

### Statistical analysis

The results are shown as the mean ± standard error of the mean of at least triplicate determinants. Student’s *t*-test was used for the analysis. A *p* value < 0.05 was considered to be significant.

## Results

**Fig. 1:**
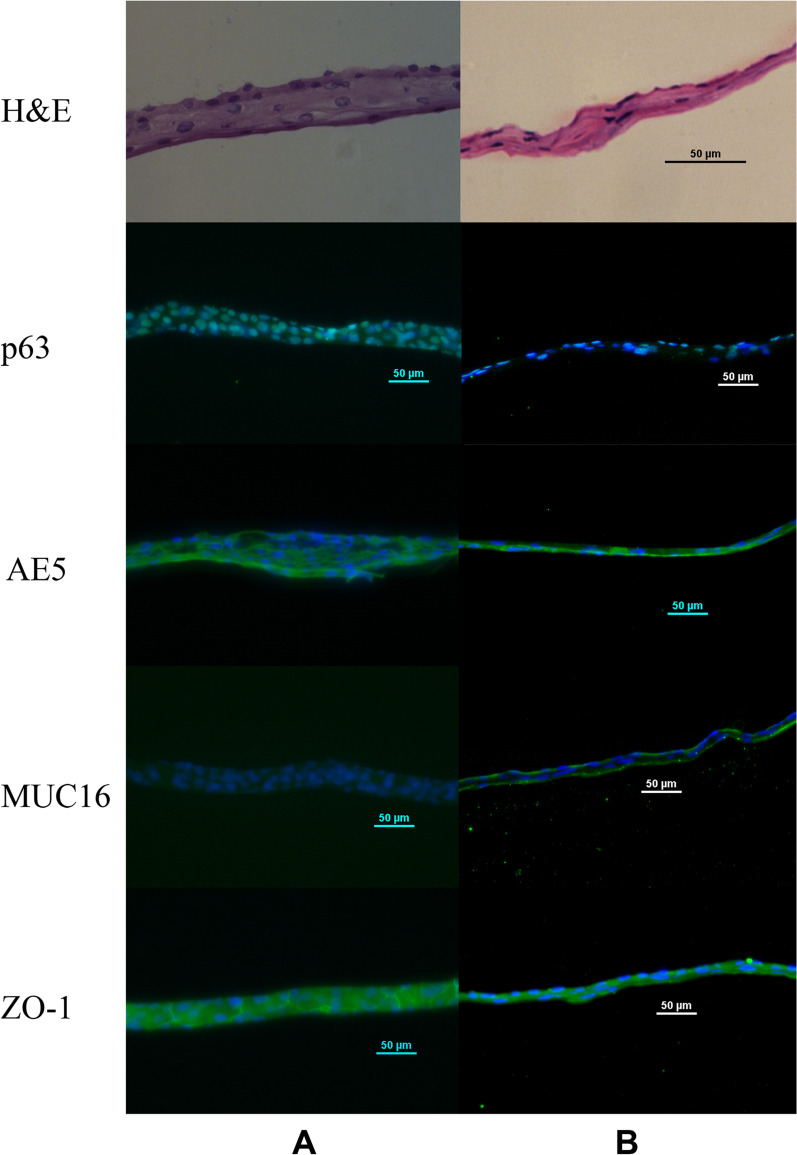
Hematoxylin and eosin (*H&E*) staining (objective lens: 40×) and immunofluorescence staining (objective lens: 20×) for the markers of epithelial stem/progenitor cells (tumor protein p63 [*p63*]), corneal differentiation (cytokeratin 3 [*AE5*]), and barrier function (membrane-anchored mucin-16 [*MUC16*] and tight junction protein-1 [ZO-1]) in the epithelial cell sheets prepared from the oral mucosal cells from subject #1 (**A**) and #3 (**B**)

**Fig. 2:**
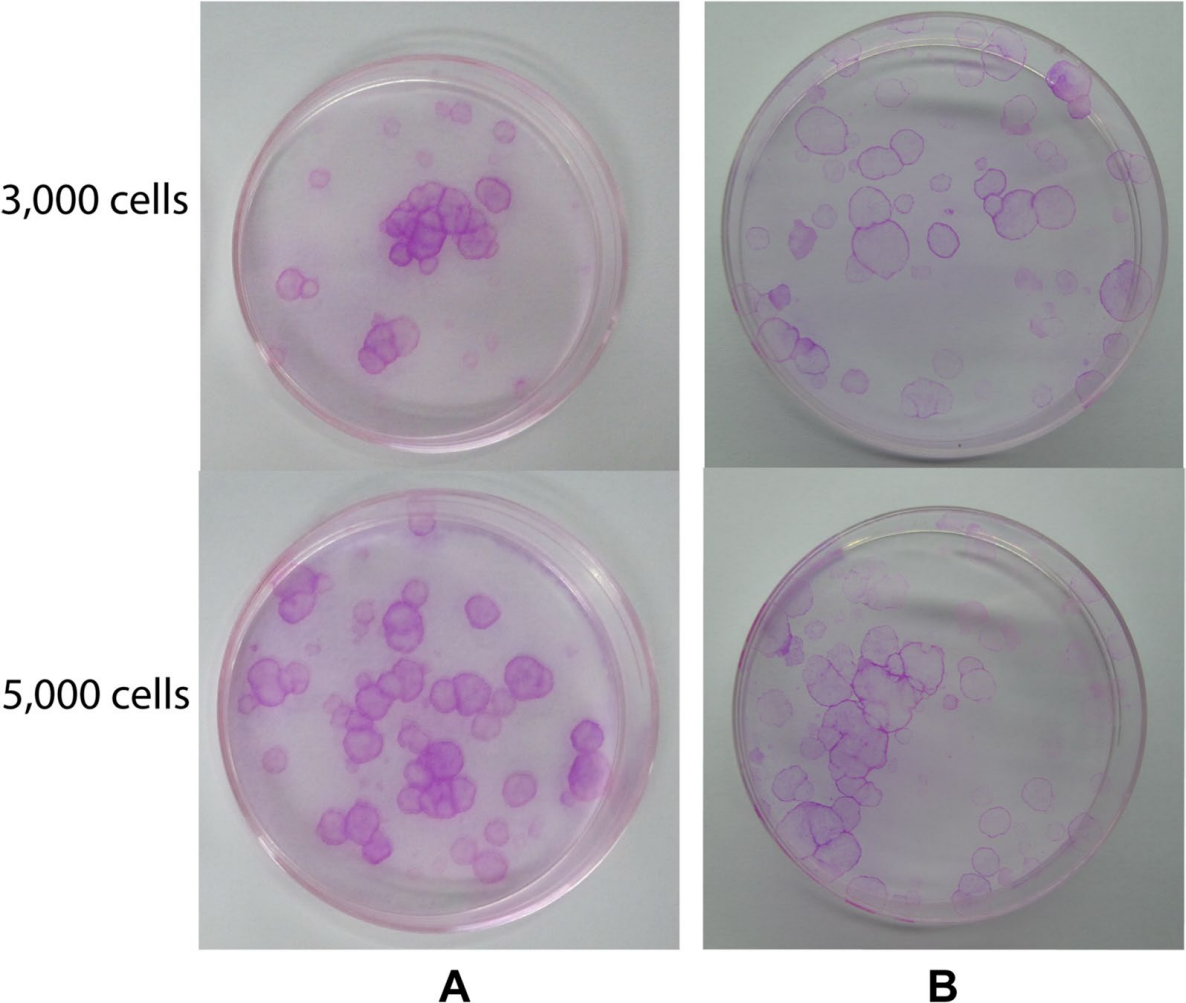
The colony-forming assay of the oral mucosal epithelial cells from subjects #1 (**A**) and #3 (**B**)

The freshly obtained oral mucosal cells together with the resulting cell sheets were evaluated physically and quantitatively (Table 2). The differentiation status of the cell sheets was determined using immunofluorescence staining with the respective antibodies (Fig 1; Table 3). The epithelial cell sheets from all patients expressed tumor protein p63 (p63), the marker of epithelial stem cells. The presence of cytokeratin 3 (AE5), the unique marker of corneal epithelium, was clearly observed in subjects 1, 2, and 3, but was faint in patients 4, 5, and 6. Likewise, the presence of ZO-1, the epithelial tight junction protein 1, was also clearly observed in patients 1, 2, and 3. The proliferative activity of all cell sheets was confirmed using the CFA (Fig. 2; Table 4).

**Table 2:** Characteristics of all patients and their respective epithelial cell sheets

Subject characteristics	Subjects					
	#1	#2	#3	#4	#5	#6
Age (years)	37	59	66	37	34	44
Sex	Female	Male	Male	Female	Female	Male
Eye	Right	Right	Left	Right	Right	Left
Eye disease	Acid burn	SJS	Alkali burn	SJS	SJS	Chemical burn (Thinner)
Obtained oral mucosal cells (*n*)	32.2 × 10^5^	14.9 × 10^5^	21.1 × 10^5^	23.2 × 10^5^	27.7 × 10^5^	27.2 × 10^5^
Mucosal cell viability (%)	71.6	63.0	86.5	76.6	72.8	75.8
Seeded cells (*n*)	3.0 × 10^5^	3.0 × 10^5^	3.0 × 10^5^	5 × 10^5^	2.0 × 10^5^	2.0 × 10^5^
Cell sheet (day)	13	13	12	13	14	14
Total cells/sheet (*n*)	17.6 × 10^5^	12.7 × 10^5^	7.4 × 10^5^	6 × 10^5^	11.3 × 10^5^	3.35 × 10^5^
Sheet viability (%)	92.1	85.6	83.1	74.4	74.1	70.7
Epithelial cell purity (%)	98.3	98.3	95.6	96.5	95.8	95.0

*SJS* Stevens-Johnson syndrome

**Table 3:** The mean fluorescence intensity of the markers of differentiation in corneal epithelial cells using ImageJ software

Markers	Subjects						Average	Standard deviation
	#1	#2	#3	#4	#5	#6		
p63	2.09	0.94	2.52	1.66	0.63	0.70	1.42	0.79
AE5	18.91	1.88	11.23	14.11	8.44	7.80	10.40	5.83
ZO-1	2.71	2.85	4.22	4.63	3.81	1.91	3.35	1.03
MUC16	4.74	1.22	1.30	1.25	0.94	5.03	2.42	1.92

*p63* Tumor protein 63,* AE5* anti-cytokeratin 3 mAb,* MUC16* mucin-16,* ZO-1* tight junction protein-1

Values in table are presented as arbitrary units based on immunofluorescence intensity

**Table 4:** Results of the colony-forming assay of the seeded oral mucosal epithelial cells at the indicated cell density

Subjects	Colony-forming assay (%)				Average	Standard deviation
	3 × 10^3^ seed cells		5 × 10^3^ seed cells			
#1	1.40	1.50	1.50	1.74	1.54	0.14
#2	1.77	1.87	1.68	1.60	1.73	0.11
#3	2.37	1.30	1.86	1.80	1.83	0.44
#4	0.80	0.83	0.54	0.72	0.72	0.13
#5	6.00	5.83	5.14	4.88	5.46	0.54
#6	4.73	5.20	4.16	3.96	4.51	0.56

**Fig. 3:**
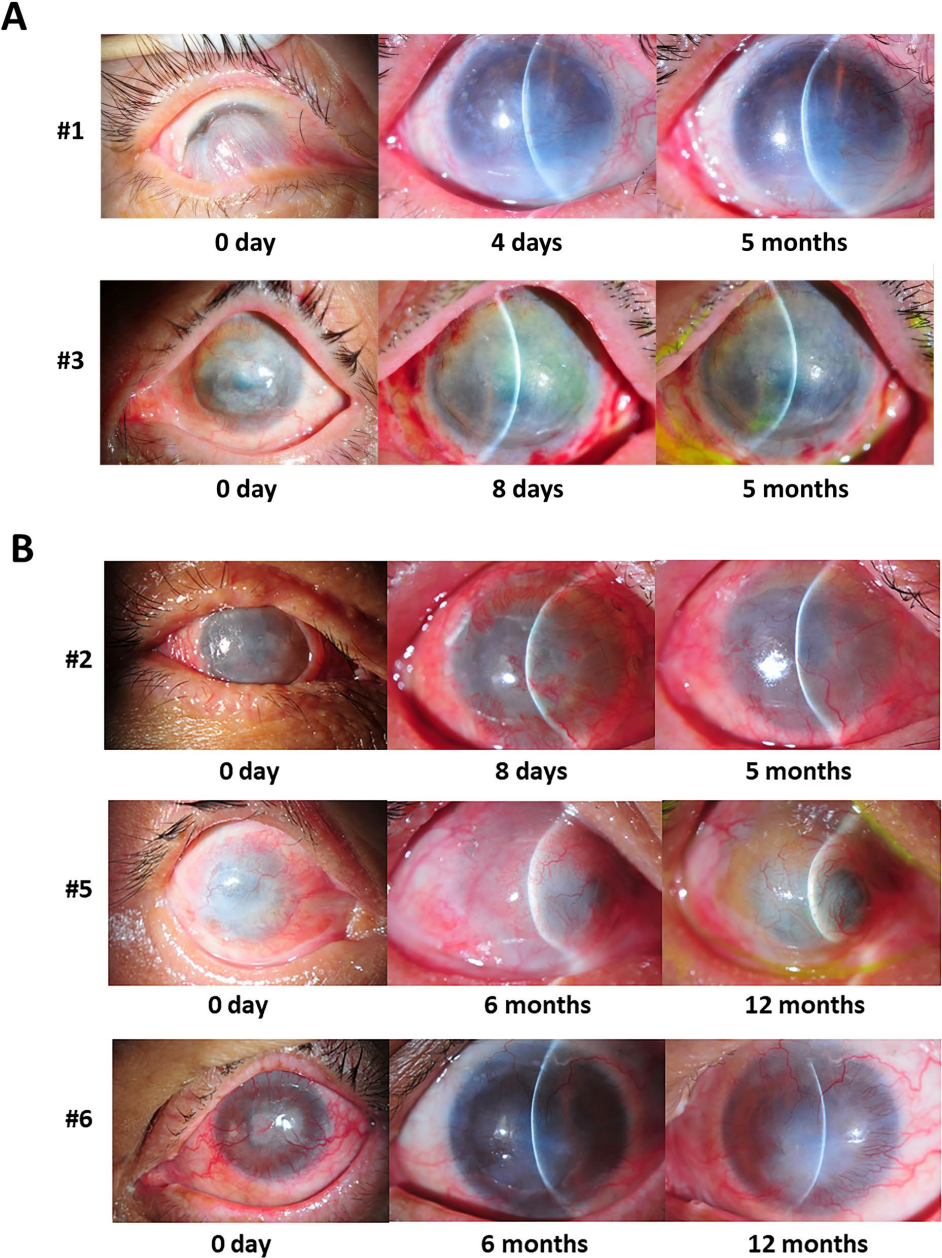
Ophthalmic examinations on the inflicted eyes from successful responders (**A**, subjects #1 and #3) and less successful responders (**B**, subjects #2, #5, and #6) were taken before the operation and after the operation at the designated time points

The scoring of clinical outcomes and severity (Table 5) was applied throughout the study (Tables 6, 7, 8, 9, 10, 11). The physical appearance of the affected eyes of successful responders (subjects #1 and #3; Fig. 3a) and less successful responders (subjects #2, #5, and #6; Fig. 3b) was assessed before and after the operation. The transplanted grafts were in good condition within a few days after the operation until at least 5 months post-surgery. At 2 weeks post-surgery, visual acuities had improved in 5 patients (Table 7). Two patients (subjects #5 and #6) had corneal ulcer requiring systemic and topical antibiotics. Subject #5 needed to stop wearing contact lenses in the first month post-surgery while subject #6 stopped wearing contact lenses at 4 months post-surgery. Subjects #4 lost her contact lenses with no corneal infection and stopped taking medication before the end of the study. At the 1-year postoperative check-up, the grading of tissue transplantation was excellent in two patients (subjects #1 and #3) who had chemical injuries (Table 11). The visual outcome of four patients (subjects #1, #2, #3, #5) along the post-operative course.

**Table 5:** Clinical grading and severity scoring

Clinical outcomes	Clinical grading and severity scoring			
	0	1	2	3
Corneal opacity	Iris detail clearly visualized	Partial obscured	Poorly seen	Completely obscured
Neovascularization	No	Periphery	Extend to pupil	Beyond pupil
Keratinization	No	< 1/4	1/4–1/2	> 1/2 Cornea
Conjunctival hyperemia	No	Sectoral engorgement	diffuse	Severe
Symblepharon	No	Conjunctival surface	< 1/2 Cornea	> 1/2 Cornea
Superficial punctate keratitis	A_1_ D_1_	A_1_ D_2_, A_2_ D_1_	A_1_ D_3_, A_2_ D_2,_ A_3_ D_1_	A_2_ D_3_, A_3_ D_2,_ A_3_ D_3_
Corneal epithelial defect	No	< 1/4	1/4–1/2	> 1/2 Cornea
Conjunctivalization	No	< 1/4	1/4–1/2	> 1/2 Cornea
Subjective symptom	No	Mild	Moderate	Severe
Corneal infection	No	Require eye drop	Require systemic	Require surgery
Endophthalmitis	No	Present	–	–

Superficial punctate keratitis grading scale is based on the sum of the area (A) and density (D) grades measured using an anterior fluorophotometer

**Table 6:** Clinical characteristics (preoperative/day 0)

Clinical characteristics	Subjects					
	#1	#2	#3	#4	#5	#6
Visual acuity	Hand motion	Hand motion	6/60	Count finger 1 foot	Light perception	Count finger 1/2 foot
Corneal opacity	3	1	3	2	3	1
Neovascularization grade	3	3	3	3	3	3
Tear Schirmer I without anesthesia	0	0	3	0	15	0
Keratinization	0	1	0	1	0	0
Conjunctival hyperemia	3	3	1	1	3	1
Symblepharon	3	1	3	1	3	0
Keratitis	N/A	0	0	0	N/A	2
Corneal epithelial defect	0	0	0	0	0	2
Conjunctivalization	3	3	3	3	3	3
Pain, irritation	1	2	1	1	1	1

*N/A* Not applicable

**Table 7:** Clinical characteristics (2 weeks postoperative)

Clinical characteristics	Subjects					
	#1	#2	#3	#4	#5	#6
Visual acuity	Fc1ʹ	Fc1ʹ	6/96	6/48	Hand motion	Fc ½ʹ
Pinhole	Fc3ʹ	Fc1ʹ	6/96	6/19	Hand motion	Fc ½ ʹ
Corneal opacity	1	1	2	1	2	1
Neovascularization grade	3	3	3	2	3	1
Tear Schirmer I	32	16	–	8	–	23
Keratinization	0	0	0	0	0	0
Conjunctival hyperemia	1	3	1	2	3	2
Symblepharon	0	0	0	1	1	0
Keratitis	1	1	0	0	2	0
Corneal epithelial defect	3	1	1	2	3	2
Conjunctivalization	0	0	0	0	3	0
Pain, irritation	1	1	1	1	1	2
Corneal infection	0	0	0	0	3 (*Pseudomona*s)	2 (*Staphylococcus coagulase* negative)
Endophthalmitis	0	0	0	0	0	0

*Fc* Finger count

**Table 8:** Clinical characteristics (1 month postoperative)

Clinical characteristics	Subject.					
	#1	#2	#3	#4	#5	#6
Visual acuity	Fc1ʹ	Fc1ʹ	6/60	6/60	Hand motion	Fc ½ʹ
Pinhole	Fc3ʹ	Fc1ʹ	6/60	6/38^−2^	Hand motion	Fc ½ʹ
Corneal opacity	1	1	1	1	2	2
Neovascularization grade	3	3	3	1	3	1
Tear Schirmer I	>35	14	–	17	35	–
Keratinization	0	0	0	0	0	0
Conjunctival hyperemia	1	2	1	1	3	2
Symblepharon	0	0	0	1	1	0
Keratitis	1	0	0	0	N/A	1
Corneal epithelial defect	2	0	0	2	3 (corneal thinning)	1
Conjunctivalization	0	0	0	0	3	1
Pain, irritation	1	1	1	1	1	2
Corneal infection	0	0	0	0	0	1
Endophthalmitis	0	0	0	0	0	0

*Fc* Finger count, *N/A* Not applicable

**Table 9:** Clinical characteristics (3 months postoperative)

Clinical characteristics	Subjects					
	#1	#2	#3	#4	#5	#6
Visual acuity	Fc1ʹ	Fc1/2ʹ	6/60	Hand motion	Hand motion	–
Pinhole	Fc1ʹ	Fc1/2ʹ	6/60	Hand motion	Hand motion	–
Corneal opacity	1	3	1	2	3	–
Neovascularization grade	3	3	3	3	3	–
Tear Schirmer I	19	0	-	0.5	10	–
Keratinization	0	0	0	0	0	–
Conjunctival hyperemia	1	3	1	1	3	–
Symblepharon	1	0	0	1	1	–
Keratitis	0	2	0	1	N/A	–
Corneal epithelial defect	0	0	0	0	0	–
Conjunctivalization	0	3	0	3	3	–
Pain, irritation	1	2	1	1	1	–
Corneal infection	0	0	0	0	0	–
Endophthalmitis	0	0	0	0	0	–

*Fc* Finger count, *N/A* Not applicable

**Table 10:** Clinical characteristics (6 months postoperative)

Clinical characteristics	Subject					
	#1	#2	#3	#4	#5	#6
Visual acuity	Fc1ʹ	Fc1/4ʹ	6/60	Hand motion	Hand motion	Fc1/2ʹ
Corneal opacity	1	3	3	3	3	1
Neovascularization grade	3	3	0	3	3	1
Tear Schirmer I	26	1	0	2	11	0
Keratinization	0	0	0	0	0	0
Conjunctival hyperemia	1	3	1	2	3	2
Symblepharon	1	1	0	1	1	0
Keratitis	0	0	0	1	N/A	1
Corneal epithelial defect	0	0	0	0	0	0
Conjunctivalization	0	3	0	3	3	3
Pain, irritation	1	1	0	2	1	1
Corneal infection	0	0	0	0	0	0
Endophthalmitis	0	0	0	0	0	0

*Fc* Finger count, *N/A* Not applicable

**Table 11:** Clinical characteristics (1 year postoperative)

Clinical characteristics	Subject.					
	#1	#2	#3	#4	#5	#6
Visual acuity	Fc1ʹ	Fc1ʹ	6/192	Fc1ʹ	Hand motion	Fc1/2ʹ
Corneal opacity	1	3	3	3	3	1
Neovascularization grade	3	3	0	3	3	1
Tear Schirmer I	17	0.5	0	3	10	0
Keratinization	0	0	0	0	0	0
Conjunctival hyperemia	1	3	1	1	2	2
Symblepharon	1	1	0	1	1	0
Keratitis	0	1	0	1	N/A	1
Corneal epithelial defect	0	0	0	0	0	0
Conjunctivalization	0	3	0	3	3	3
Pain, irritation	1	1	0	1	1	1
Corneal infection	0	0	0	0	0	0
Endophthalmitis	0	0	0	0	0	0
Overall grading/rating	5 excellent	3 good	5 excellent	1 poor	1 poor	1 poor

*Fc* Finger count, *N/A* Not applicable

## Discussion

The cultivated oral mucosal epithelial sheet requires expertise in cell culture, with temperature control and efficiency, including careful transplantation. Anti-inflammatory medicine, such as corticosteroid, is needed to maintain cell recovery, decrease fibrous adhesion (symblepharon), and promote healing with artificial tears. The result is critical at each evaluation time point to avoid the side effects of the corticosteroid treatment, especially infection. In cell culture preparation, the viability of cells from subjects with acid burn (92.1%) was higher than that from subjects with SJS and others. Chemical injuries that involve only the eyes, not the mouth, may result in better cell viability. Most patients in our study had severe dry eyes, neovascularization grade 3, and symblepharon.

At 1 year post-surgery, vision had improved compared to the pre-operation condition, there was more tear production, minimal symblepharon, and not corneal epithelial defect. The excellent grading result at 1 year was achieved in two patients with chemical burn. To the contrary, those with SJS may have had oral involvement that resulted in a lower number of viable mucosal epithelial cells, of which the viability may also have been lower. All of these patients had severe dry eyes and a higher risk of infection that ended up with a fair result. Tears of the severe chronic SJS eyes contained cytokines (interleukin-8 and granzyme B) [17] that reflected an ongoing immune reaction. The presence of both of these cytokines in the tears of patients with SJS could induce angiogenesis and cytotoxicity in the graft. Both the presence of these cytokines and the impaired treatment regimen could contribute towards the unsuccessful outcome in these patietns. Postoperative management required long-term usage of anti-inflammatory drug with different regimens and variations to prevent infection. Any recurring inflammation would result in increasing fibrosis.

## Conclusions

Cultivated oral mucosal epithelial cell sheet transplantation was successful in the treatment of eyes with chemical injury at 1 month post-surgery. Long-term management and follow-up are required for all patients who need to strictly adhere to the instructions to achieve the optimal result of transplantation.

## Data Availability

The dataset supporting the conclusions of this article is included within the article.

## Abbreviations

CFA: Colony-forming assay
LSCD: Limbal stem cell deficiency
SJS: Stevens-Johnson Syndrome
